# Tetrodotoxin-insensitive electrical field stimulation-induced contractions on *Crotalus durissus terrificus* corpus cavernosum

**DOI:** 10.1371/journal.pone.0183766

**Published:** 2017-08-24

**Authors:** Rafael Campos, Fabíola Z. Mónica, Renata Lopes Rodrigues, Julio Alejandro Rojas-Moscoso, Ronilson Agnaldo Moreno, José Carlos Cogo, Marco Antonio de Oliveira, Edson Antunes, Gilberto De Nucci

**Affiliations:** 1 Faculty of Medical Sciences, Department of Pharmacology, University of Campinas (UNICAMP), Campinas, Brazil; 2 Faculty of Health Sciences, Department of Pharmacology, FACISB, Barretos, Brazil; 3 Faculty of Medical Sciences, Brazil University, Fernandópolis, Brazil; 4 Institute of Biomedical Sciences, University of São Paulo (USP), São Paulo, Brazil; University of Calgary, CANADA

## Abstract

Reptiles are the first amniotes to develop an intromitent penis, however until now the mechanisms involved in the electrical field stimulation-induced contraction on corpora cavernosa isolated from *Crotalus durissus terrificus* were not investigated. Crotalus and rabbit corpora cavernosa were mounted in 10 mL organ baths for isometric tension recording. Electrical field stimulation (EFS)-induced contractions were performed in presence/absence of phentolamine (10 μM), guanethidine (30 μM), tetrodotoxin (1 μM and 1mM), A-803467 (10 μM), 3-iodo-L-Tyrosine (1 mM), salsolinol (3 μM) and a modified Krebs solution (equimolar substitution of NaCl by N-methyl–D-glucamine). Immuno-histochemistry for tyrosine hydroxylase was also performed. Electrical field stimulation (EFS; 8 Hz and 16 Hz) caused contractions in both Crotalus and rabbit corpora cavernosa. The contractions were abolished by previous incubation with either phentolamine or guanethidine. Tetrodotoxin (1 μM) also abolished the EFS-induced contractions of rabbit CC, but did not affect EFS-induced contractions of Crotalus CC. Addition of A-803467 (10 μM) did not change the EFS-induced contractions of Crotalus CC but abolished rabbit CC contractions. 3-iodo-L-Tyrosine and salsolinol had no effect on EFS-induced contractions of Crotalus CC and Rabbit CC. Replacement of NaCl by N- Methyl-D-glucamine (NMDG) abolished EFS-induced contractions of rabbit CC, but did not affect Crotalus CC. The presence of tyrosine hydroxylase was identified in endothelial cells only of Crotalus CC. Since the EFS-induced contractions of Crotalus CC is dependent on catecholamine release, insensitive to TTX, insensitive to A803467 and to NaCl replacement, it indicates that the source of cathecolamine is unlikely to be from adrenergic terminals. The finding that tyrosine hydroxylase is present in endothelial cells suggests that these cells can modulate Crotalus CC tone.

## Introduction

Penile erection is a neurovascular event dependent on cavernosal smooth muscle relaxation and elevation of local flux of blood [1,2]. In mammals, the main physiological component involved in the detumescent state is the liberation of catecholamine by adrenergic nerves, inducing cavernosal muscle contraction. Nitric oxide (NO) is the major component responsible for initiating and maintaining the tumescent state, by promoting cavernosal smooth muscle relaxation [3–5].

Sodium voltage-gated channels (VGSC) are important ion channels involved in nerve depolarization [6]. Treatment with tetrodotoxin (TTX) or other inhibitors of VGSC abolishes the nitrergic relaxation induced by electrical field stimulation (EFS) in rabbit, monkey and human corpora cavernosa preparations [7–9]. In Crotalus corpus cavernosum (CCC), the EFS-induced relaxation is not affected by TTX [10], indicating the possible presence of a TTX-insensitive sodium channel. The purpose of this study was to characterize the transmural EFS-induced contractions in CCC.

## Material and methods

### Animals

All experimental procedures using *Crotalus durissus terrificus*, New Zealand white rabbit and the *Callithrix jachus* (marmoset) were approved by the Institutional Animal Care and Use Committee of the University of Campinas (Committee for Ethics in the Use of Animals- CEUA/UNICAMP: protocol numbers 1655–1, 2720–1 and 3811–1, respectively) and were performed in accordance with the Ethical Principles for Animal Research adopted by the Brazilian College for Animal Experimentation.

The use of *Crotalus durissus terrificus* and *Callithrix jachus* was authorized by the Brazilian Institute for Environment (Sisbio: 18020–1 and Sisbio 16951–1, respectively). Male *Crotalus durissus terrificus* (body weight: 400–750g) were provided by the Serpentarium of the Center for the Study of Nature at the University of Vale do Paraiba (UNIVAP, São José dos Campos, SP, Brazil). Male New Zealand rabbits (3.5–4 Kg) were provided by Granja RG (Suzano, SP, Brazil) and maintained in the multidisciplinary center for biological investigation on laboratory animal science (CEMIB). *Callithrix jachus* (270–320 g) were provided by Parque Ecológico Tietê (São Paulo, Brazil)

### Chemical and reagents

Guanethidine, phentolamine, phenylephrine, noradrenaline, N-Methyl-D-glucamine, tetrodotoxin and 3-iodo-L-Tyrosine were purchased from Sigma-Aldrich Chemicals Co. (Missouri, USA). A-803467 and salsolinol were bought from Tocris Bioscience (Bristol, UK). Chicken anti-tyrosine hydroxylase and goat polyclonal secondary antibody to chicken IgY—H&L (Alexa Fluor® 594) were acquired from Novus Biologicals (Colorado-USA) and Abcam (Massachusetts, USA), respectively.

### Corpora cavernosa preparation

The snakes, the rabbits and the monkeys were killed with isoflurane inhalation followed by ketamine (70 mg/kg) administration (intracelomatic route of administration in the snakes and intramuscular route in the rabbits and monkeys). The corpora cavernosa were removed and immediately placed in Krebs solution at 27°C for CCC and 37°C for the rabbit CC. Subsequently, four strips were obtained and were suspended vertically between two metal hooks in 10 mL organ baths containing Krebs solution (mM) NaCl (118), KCl (4.7), CaCl_2_ (2.5), MgSO_4_ (1.2), NaCO_3_ (25), KH_2_PO_4_ (1.2) Glucose (5.6) gassed with a mixture of 95%O_2_: 5% CO_2_ (pH 7.4) at 27°C and 37°C, respectively [10]. In some experiments, a modified Krebs solution (equimolar substituition of NaCl by N-methyl- D- glucamine (NMDG).

### Functional protocols

Crotalus corpora cavernosa (CCC) and rabbit corpora cavernosa (RbCC) were stretched to 5 mN and 10 mN of tension, respectively, during 45 minutes (period of stabilization) [11]. CCC and RbCC were submitted to EFS (50 V for 10 seconds and 50 V for 10 seconds, subsequently, at 8 and 16 Hz in square-wave pulses; 0.5 ms pulse width; 0.2 ms delay) using a Grass S88 stimulator (Astro- Medical, Industrial Park, RI, USA). Snake Skeletal smooth muscle preparations were electrically stimulated (8 V, 1 Hz, 0.2 ms). EFS-induced contractions were performed in presence and absence of phentolamine (10 μM), guanethidine (30 μM), tetrodotoxin (TTX 1μM and 1mM), 3-iodo-L-Tyrosine (1 mM), salsolinol (3 μM) and A-803467 (10 μM).

### Immunohistochemistry for tyrosine hydroxylase of Crotalus CC and brain and monkey CC

After macroscopic examination, the spiny regions were separated from the non-spiny regions (apical region) and fragments of 1mm^3^ were fixed in 4% paraformaldehyde, 0.1% glutaraldehyde diluted in sodium 0.1M cacodylate buffer, pH 7.2 for 60 minutes. The fragments were washed three times in 0.1M cacodylate pH 7.2 for fixative removing. Fragments were dehydrated by ethanol solution (30%-90%) and embedded in LR White medium (according to manufacture instructions, Polyscience, Inc.). Blocks of LR White containing fragments of tissue were sectioned (70–90 ηm) in ultramicrotome (Ultracut, Leica), the sections were collected on nickel grids. For fluorescence microscopy the fragments were fixed only in 4% formaldehyde in 0.1M PBS, pH 7.2, for 1 hour, after which they were washed in 0.1M PBS pH 7.2, frozen in mounting medium, sectioned by cryostat at -20°C, and collected onto glass slides. After sectioning, the slices on glass or on grids were treated as follows: they were incubated with ammonium chloride (50 mM; for 30 minutes at 25°C) followed by 3% BSA in 0.1M phosphate buffer saline (PBS) pH 7.2. Primary antibody (chicken anti-tyrosine hydroxylase, diluted 1:50, Novus Biologicals) were diluted in PBS solution + 1% BSA and incubated for 48 hours at 4° C after the antibody was removed by washing 3 times (30 min each) in 1%BSA/0.1M PBS pH 7.2. Secondary antibody (goat polyclonal diluted 1:100, Abcam) was incubated for 24 hours at 4°C and washed as described previously.

### Skeletal muscle preparation

The *musculus adductor mandibulae externus profundus* was isolated from the snake. Briefly, this muscle was immersed in a 5-mL organ bath containing Krebs solution at 27°C, gassed with a mixture of 95%0_2_: 5%CO_2._ and then electrically stimulated (8 V, 1 Hz, 0.2 ms) from a bipolar platinum electrode connected to a Grass stimulator [10].

### Data analysis

The contractions were calculated as mili-Newtons and expressed as mean ± standard error of mean of the number of experiments. To analyze the pharmacological characterization of EFS-induced contractions, two paired contractions in presence and absence of antagonists were performed, with the first stimulus being the “control” response. Data were compared using paired Student’s t test. A p value < 0.05 was considered significant.

## Results

### Evaluation of adrenergic and sodium-channel involvement on EFS-induced contractions in CCC and RbCC

**Fig 1:**
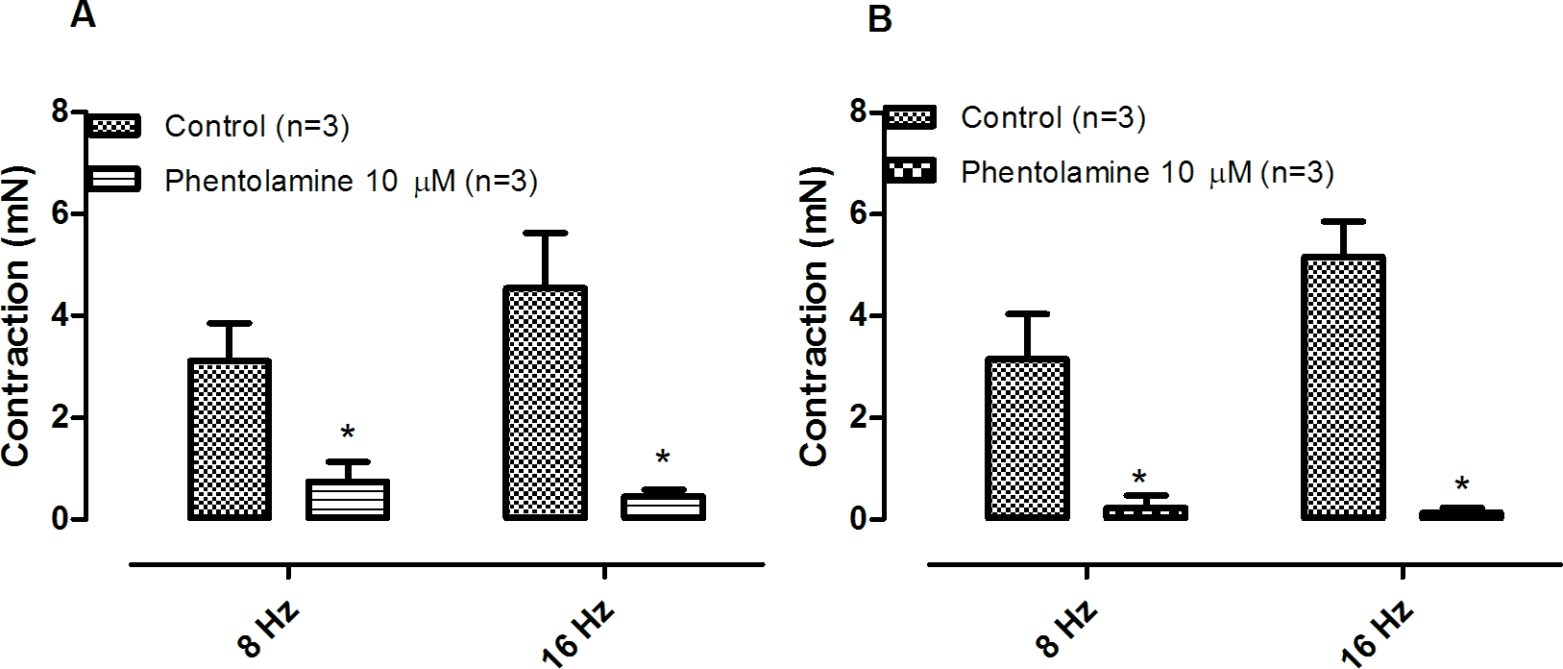
Effect of phentolamine (10 μM) on EFS-induced contractions on corpora cavernosa isolated from Crotalus **(A)** and Rabbit **(B)**. Data are expressed as mean ± standard error mean (S.E.M) paired Student's t test, * P < 0.05 vs control.

**Fig 2:**
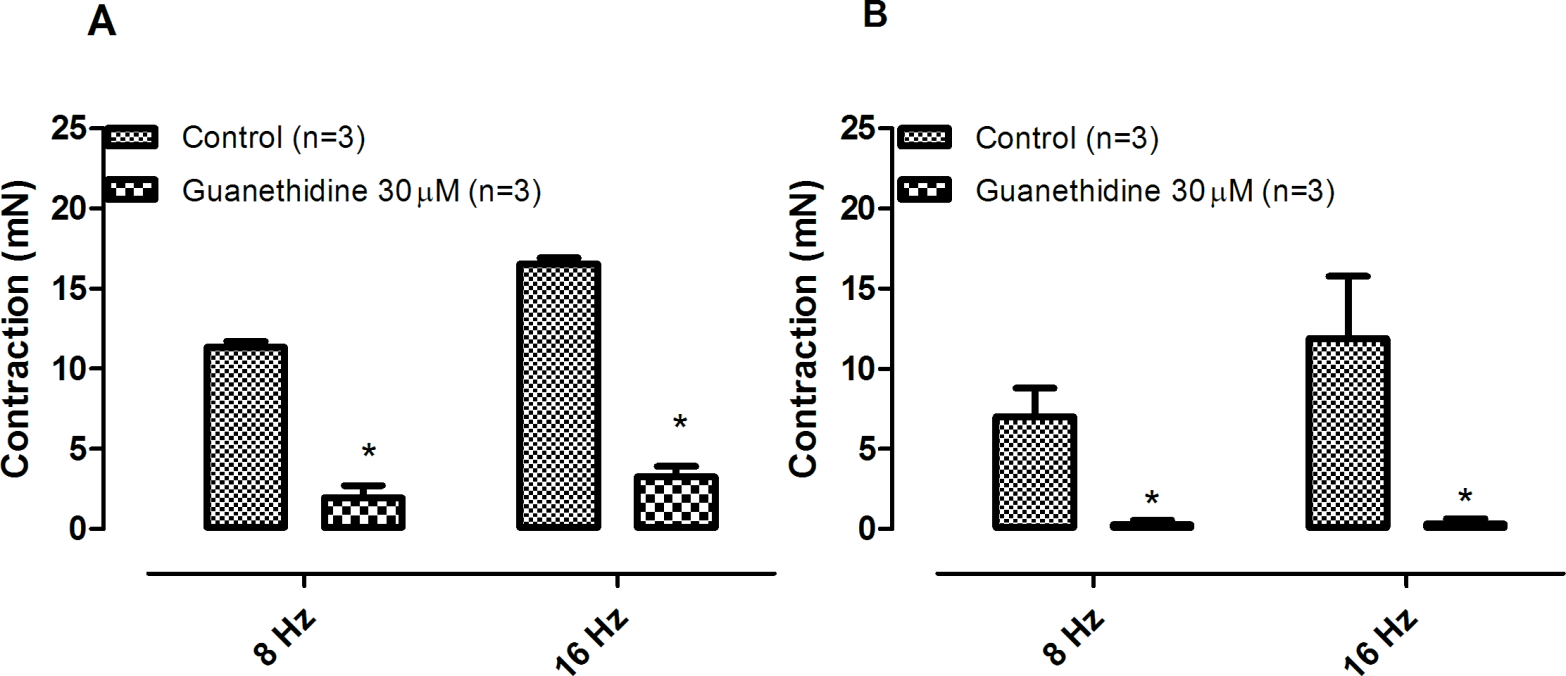
Effect of guanethidine (30 μM) on EFS-induced contractions on corpora cavernosa isolated from Crotalus **(A)** and Rabbit **(B)**. Data are expressed as mean ± standard error mean (S.E.M). * P < 0.05 vs control (paired Student's t test).

**Fig 3:**
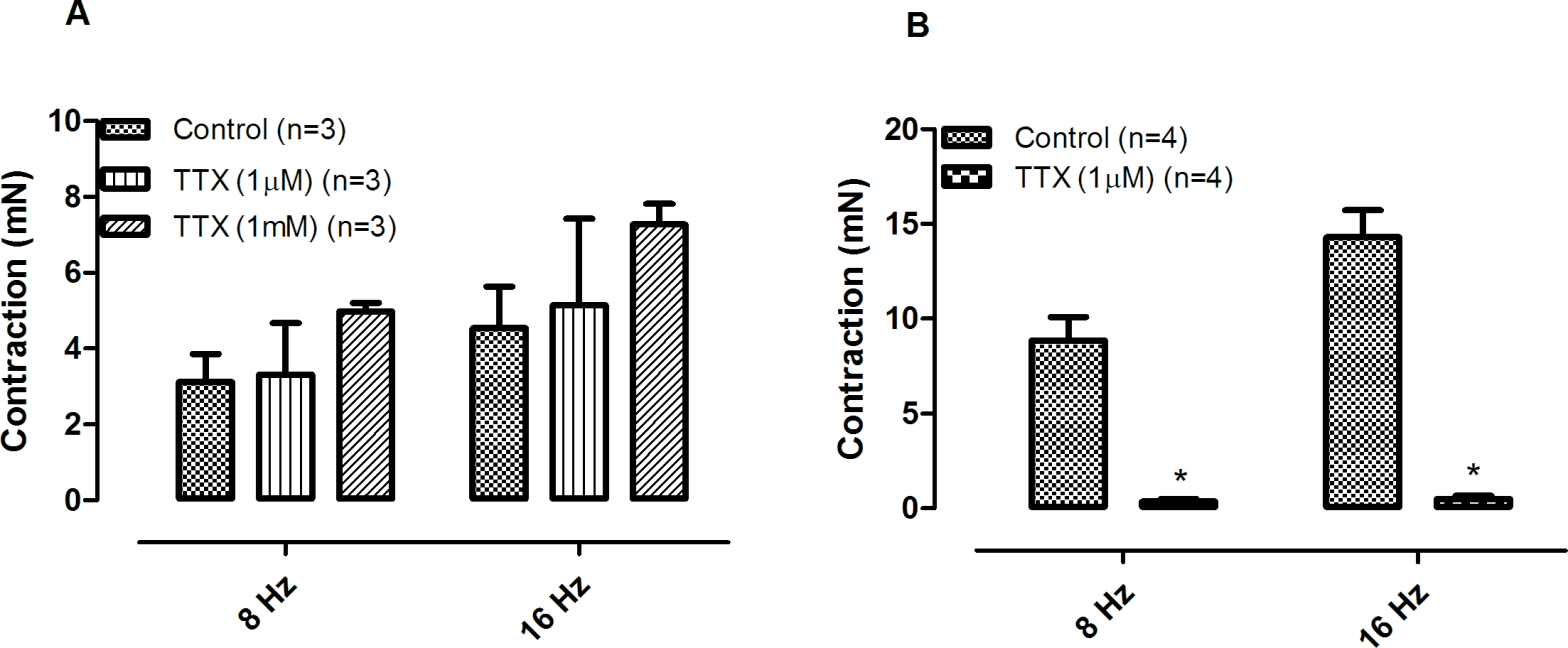
Effect of tetrodotoxin (1 μM and 1mM) on EFS-induced contractions of corpora cavernosa isolated from Crotalus **(A)** and rabbit **(B)**. Data are expressed as mean ± standard error mean (S.E.M) paired Student's t test, * P < 0.05 vs control.

**Fig 4:**
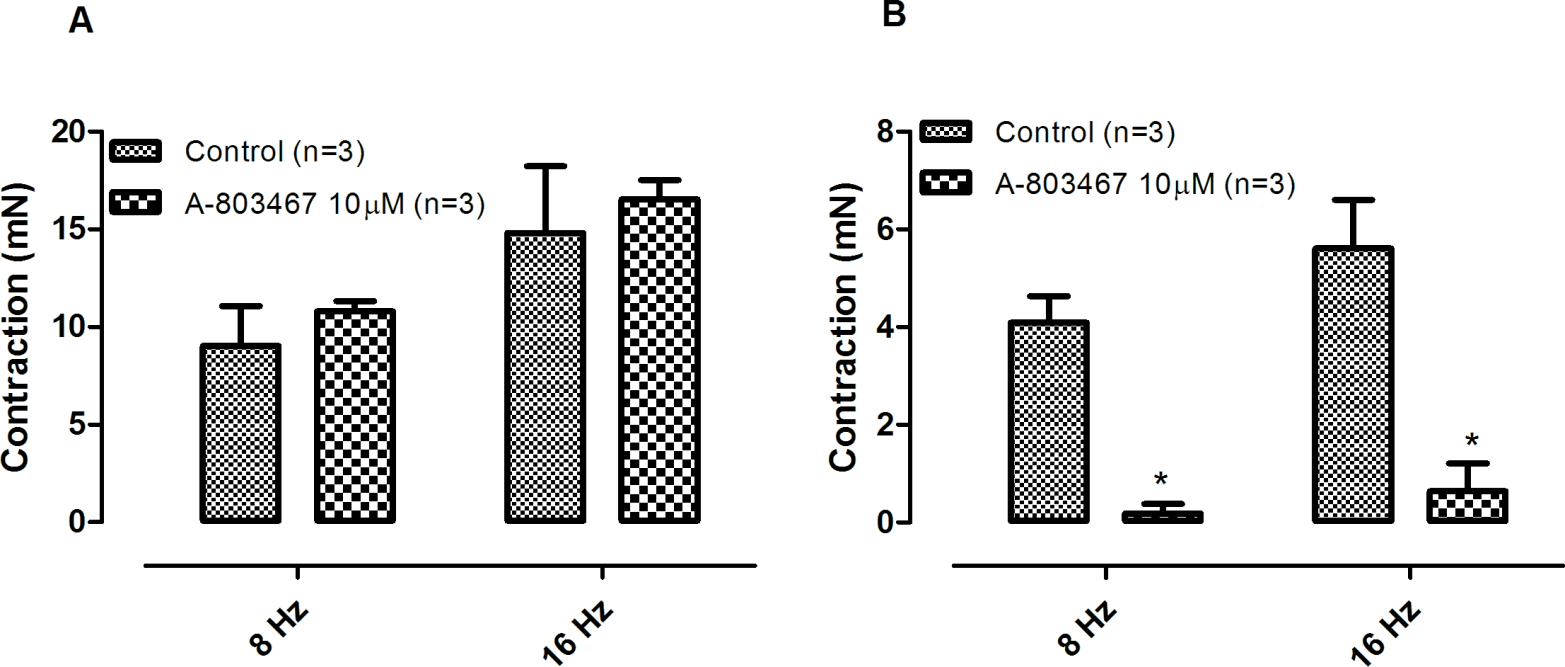
Effect of A-803467 (10 μM) on EFS-induced contractions of corpora cavernosa isolated from Crotalus **(A)** and rabbit **(B)**. Data are expressed as mean ± standard error mean (S.E.M) paired Student's t test, * P < 0.05 vs control.

**Fig 5:**
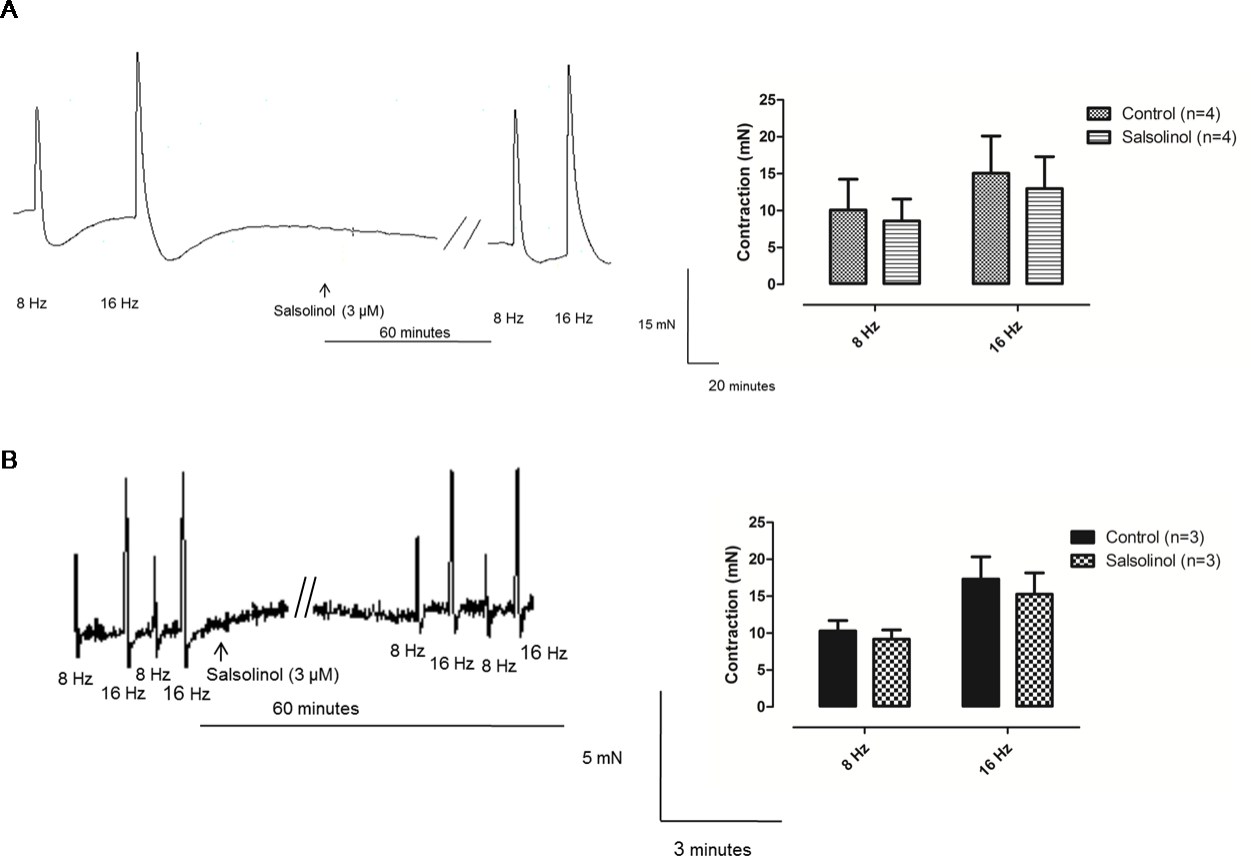
Representative illustration of EFS-induced contraction of Crotalus **(A)** and rabbit **(B)** corpora cavernosa in the presence of salsolinol (3 μM).

**Fig 6:**
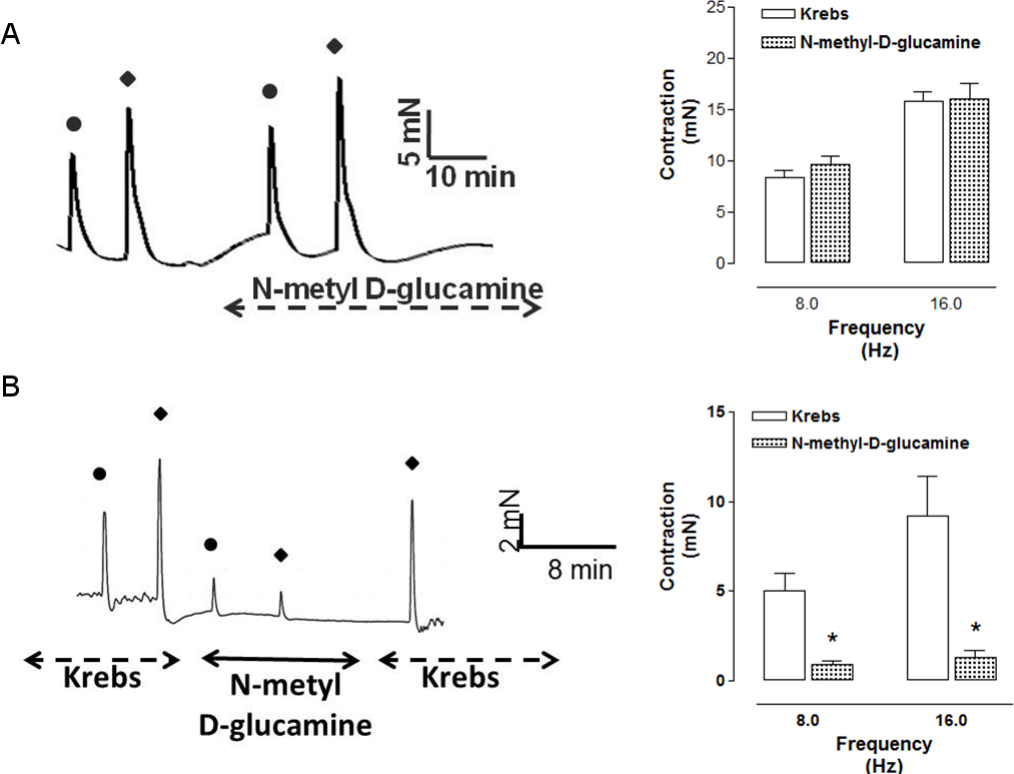
Representative illustration of EFS-induced contraction of Crotalus **(A)** and rabbit **(B)** corpora cavernosa in the presence of Kreb’s modified solution (equimolar substitution of sodium chloride by N–methyl–D-glucamine) (NMDG).

**Fig 7:**
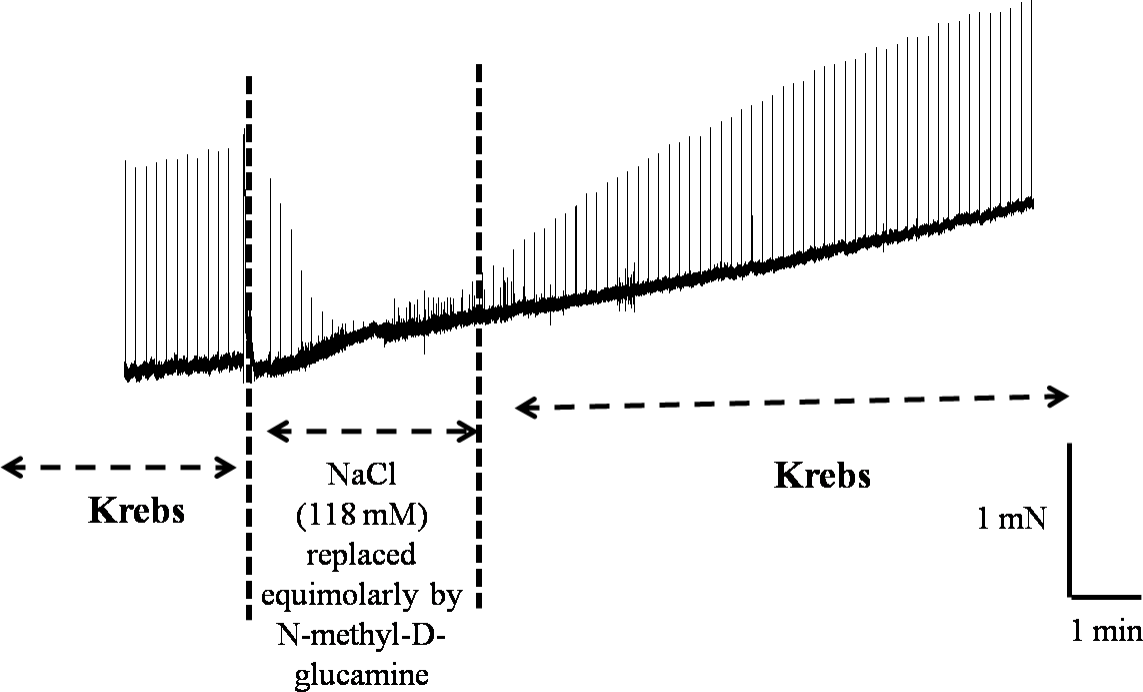
Representative illustration of EFS-induced contraction of Crotalus skeletal muscle in the presence of Kreb’s solution modified (equimolar substitution of sodium chloride by N–methyl–D-glucamine) (NMDG).

Phentolamine (10 μM) significantly reduced the EFS-induced contractions in both CCC (Fig 1A) and RbCC (Fig 1B; n = 3 for each group). Guanethidine (30 μM) significantly reduced the EFS-induced contractions in both CCC (Fig 2A) and RbCC (Fig 2B; n = 3 for each group). Electrical field stimulation (EFS; 8 Hz and 16 Hz) caused contractions in both CCC (n = 3) (Fig 3A) and RbCC (n = 4) (Fig 3B). Tetrodotoxin (1 μM and 1 mM) had no effect on the EFS-induced contractions in CCC. In contrast, Tetrodotoxin (1 μM) almost abolished EFS–induced contractions in the RbCC (n = 4). The TTX-resistant sodium channel blocker A-803467 (10 μM) did not alter the EFS-induced contractions of CCC (Fig 4A). However, it almost abolished EFS-induced contraction of RbCC (Fig 4B). The tyrosine hydroxilase inhibitor salsolinol (3 μM) had no effect in the EFS-induced contractions of both CCC (Fig 5A; n = 4) and RbCC (Fig 5B; n = 3). Similar results were obtained with another tyrosine hydroxylase inhibitor, 3-iodo-L-tyrosine (1 mM) in CCC (10.03 ± 2.62 mN; 11.49 ± 4.18 mN for 8 Hz and 15.26 ± 3.53 mN; 15.4 ± 6.6 mN for 16 Hz; n = 4) and in RbCC (1.63 ± 0.22 mN; 2.84 ± 0.55 mN for 8 Hz; 3.01 ± 0.44 mN; 5.26 ± 0.35 for 16 Hz; n = 3). Equimolar substitution of NaCl by N-methyl–D-glucamine (NMDG) did not alter the contractile response of CCC (Fig 6A), but almost abolished the EFS-induced contractions of RbCC (n = 4) (Fig 6B). The EFS-induced contractions of skeletal muscle were reduced in the presence of Krebs modified solution. (Fig 7) (n = 3).

### Immunohistochemistry

**Fig 8:**
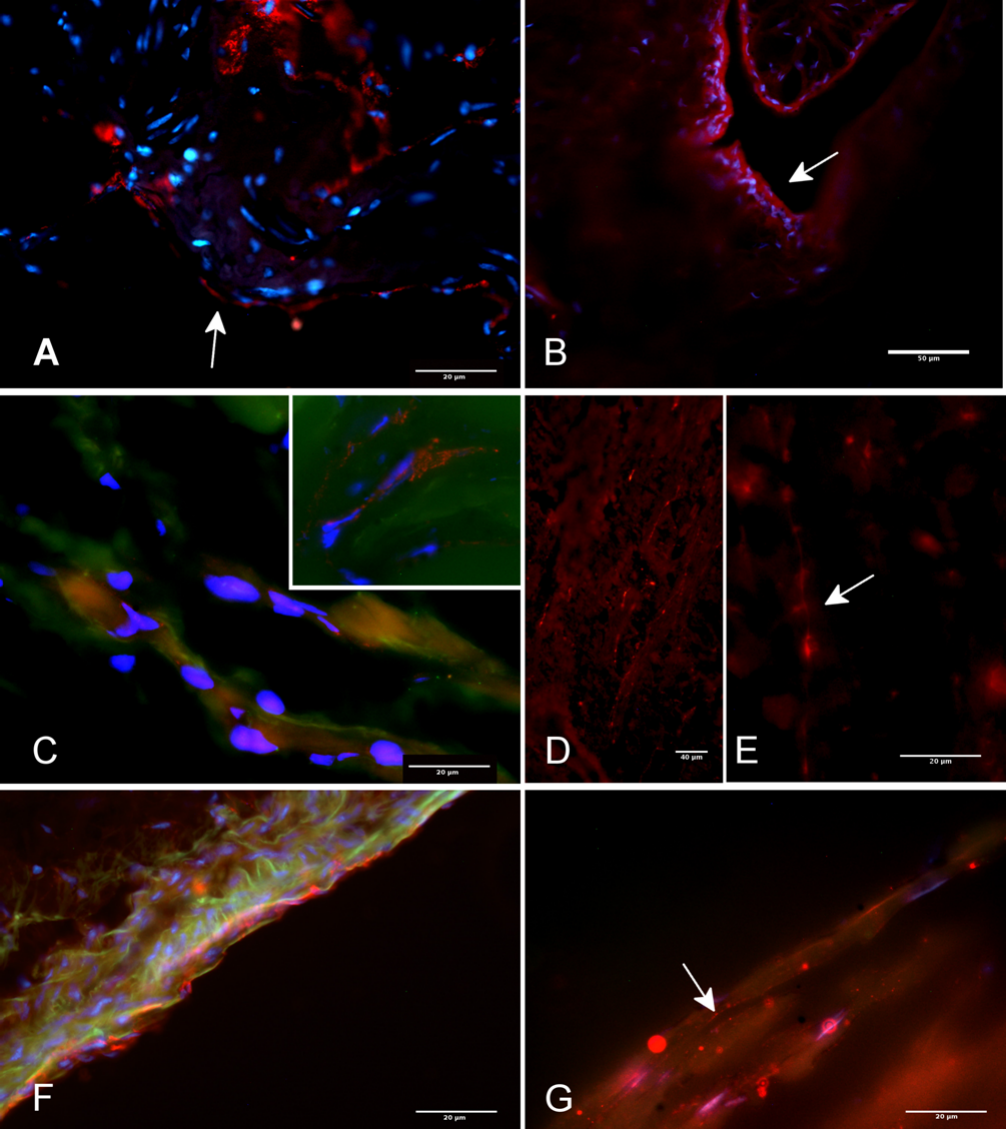
Fluorescence microscopy of tirosine hydroxylase antibody (TH Ab, red) labelled in hemipenis from *Crotallus durissus terrificus* (**A)** and in neurofilaments (green) from corpora cavernosa of *Callithrix jachus* (**C**). **B**: Endothelial layer of hemipenis from *Crotallus durissus terrificus* labelled with TH antibody. **D and E**. Brain of *Crotallus durissus terrificus* labelled with TH. **F**. Trunk region of hemipenis from *Crotalus durissus terrificus* showing TH (red) and neurofilaments (green) **G**. Apical region of hemipenis of *Crotalus*. *durissus*. *terrificus* labelled with TH Ab.

Tyrosine hydroxylase (TH) staining was observed in endothelial cells (Fig 8B; of Crotalus corpora cavernosa. TH staining was clearly observed in the neurons of Crotalus brain (Fig 8D & 8E). TH staining was observed in nerve filaments on *Callithrix jachus* corpora cavernosa (Fig 8A & 8C), but not in endothelium cells.

## Discussion

Adrenergic tone is responsible for the maintenance of the penile flaccid state, since intracavernosal injection of alpha-adrenergic antagonists such as phenoxybenzamine and phentolamine cause long-lasting erection in humans [12,13]. Electrical-field stimulation of rat [14] and rabbit [15] corpus cavernosum causes contraction and this contraction is abolished with the use of guanethidine and phentolamine, corroborating the importance of catecholamines in modulating corpus cavernosum tonus. The sodium channel blocker tetrodotoxin inhibits the EFS-induced contractions in mammalian corpora cavernosa, indicating that the catecholamine release are sensitive to sodium channel blockade [14].

The EFS-induced contractions of *Crotalus* corpora cavernosa shared some similarities with the contractions observed in mammalian corpora cavernosa, since they are also abolished with incubation of sympatholitic agents such as guanethidine or phentolamine. Interestingly, unlike mammalian CC, EFS-induced contractions of *Crotalus* CC are insensitive to TTX. This is not surprising, since EFS-induced relaxation of *Crotalus* CC has been reported to be insensitive to TTX [10]. TTX-insensitve sodium channels have been previously described in dorsal root ganglion [16], group C sensory neurons [17], denervated skeletal muscle [18] and cardiac muscle preparation [19]. TTX-insentive sodium channels have also been previously reported in skeletal muscle of the garter snake *Thamnophissirtalis* [20–22]. This has not been confirmed in *Crotalus durissus terrificus* skeletal muscle [10]. A-803467 has been described as a selective inhibitor of TTX-insensitive sodium channels in rat [23]. Interestingly, A-803467 (10 μM) had no effect on the EFS-induced contraction of Crotalus CC but almost abolished rabbit CC contractions. Whether this result may be due to lack of specificity of this sodium channel inhibitor in rabbit CC remains to be further investigated. Interestingly, the two tyrosine hydroxylase inhibitors had effect on EFS-induced contractions in neither rabbit CC nor CCC. The lack of inhibition may reflect a very efficient system of catecholamine re-uptake, as seen in other tissues [24,25].

Replacement of NaCl with N–methyl–D-glucamine caused significant inhibition of EFS-induced contraction of both rabbit CC and Crotalus skeletal muscle, as expected. However, no effect was observed in Crotalus CC, indicating that the mechanism by which EFS induces contraction of Crotalus CC is very different from that of mammalian CC. These findings would indicate that adrenergic terminal is an unlikely source for the catecholamine release-induced by EFS in *Crotalus* CC.

Immunohistochemistry for tyrosine hydroxylase, an enzyme essential for the conversion of dopamine into noradrenaline, revealed its presence in nerve terminals of the monkey *Callitrix jaccus* (Fig 8C), as it has been reported for rat [26] and human CC [27]. Using mammalian antibodies in other taxons such as reptilia tissue may present difficulties in interpretation. However, the finding that tyrosine hydroxylase was readily identified in neuronal bodies of Crotalus brain indicates that the rabbit antibody recognizes the snake tyrosine hydroxylase. Interestingly, tyrosine hydroxylase was identified only in the endothelial cells of *Crotalus* CC. Considering the above pharmacological and histological findings, it is very likely that the catecholamine release responsible for the EFS-induced contractions is not neurogenic. Interestingly, tyrosine hydroxylase is present in endothelial cells in both bovine aortic endothelial cells and mice superficial femoral arteries from hindlimbs, and its expression is increased in both hypoxia and ischemia, respectively [28]. Thus, a local catecholamine release by the endothelium may modulate local tonus in CCC.

## Conclusion

Electrical field-induced contractions in CCC is dependent on catecholamine release, but not from adrenergic terminals. Immunohistochemistry indicates the endothelium as the possible source for catecholamine release.
